# Viperin Is Induced following Dengue Virus Type-2 (DENV-2) Infection and Has Anti-viral Actions Requiring the C-terminal End of Viperin

**DOI:** 10.1371/journal.pntd.0002178

**Published:** 2013-04-18

**Authors:** Karla J. Helbig, Jillian M. Carr, Julie K. Calvert, Satiya Wati, Jennifer N. Clarke, Nicholas S. Eyre, Sumudu K. Narayana, Guillaume N. Fiches, Erin M. McCartney, Michael R. Beard

**Affiliations:** 1 School of Molecular and Biomedical Science, University of Adelaide, Adelaide, South Australia, Australia; 2 Microbiology and Infectious Diseases, School of Medicine, Flinders University, Bedford Park, Adelaide, South Australia, Australia; Florida Gulf Coast University, United States of America

## Abstract

The host protein viperin is an interferon stimulated gene (ISG) that is up-regulated during a number of viral infections. In this study we have shown that dengue virus type-2 (DENV-2) infection significantly induced viperin, co-incident with production of viral RNA and via a mechanism requiring retinoic acid-inducible gene I (RIG-I). Viperin did not inhibit DENV-2 entry but DENV-2 RNA and infectious virus release was inhibited in viperin expressing cells. Conversely, DENV-2 replicated to higher tires earlier in viperin shRNA expressing cells. The anti-DENV effect of viperin was mediated by residues within the C-terminal 17 amino acids of viperin and did not require the N-terminal residues, including the helix domain, leucine zipper and *S*-adenosylmethionine (SAM) motifs known to be involved in viperin intracellular membrane association. Viperin showed co-localisation with lipid droplet markers, and was co-localised and interacted with DENV-2 capsid (CA), NS3 and viral RNA. The ability of viperin to interact with DENV-2 NS3 was associated with its anti-viral activity, while co-localisation of viperin with lipid droplets was not. Thus, DENV-2 infection induces viperin which has anti-viral properties residing in the C-terminal region of the protein that act to restrict early DENV-2 RNA production/accumulation, potentially via interaction of viperin with DENV-2 NS3 and replication complexes. These anti-DENV-2 actions of viperin show both contrasts and similarities with other described anti-viral mechanisms of viperin action and highlight the diverse nature of this unique anti-viral host protein.

## Author Summary

Viperin is a virally induced host protein that has been previously shown to have antiviral activity against a variety of viruses. Here we have demonstrated that viperin is also anti-viral against the medically significant arbovirus, dengue virus. Viperin was able to inhibit dengue virus at the level of viral replication, and cell lines unable to produce normal levels of viperin grew the virus to higher titres. These anti-dengue effects of viperin were mediated by amino acid residues in its C-terminus, and did not require structural domains of the N-terminal region as has been previously shown by us and others for the related virus, hepatitis C virus. Viperin was also demonstrated to co-localise and interact with the dengue capsid protein on the surface of lipid droplets, as well as with the NS3 protein and viral RNA. Viperin's association with NS3 was further demonstrated to be involved in its anti-dengue activities. The anti-viral activities of viperin presented in this manuscript show both similarities and contrasts with other described anti-viral mechanisms for the protein and highlight the diverse nature of this unique anti-viral host protein.

## Introduction

The interferon (IFN) response is triggered in cells infected by RNA viruses, including members of the *Flaviviridae* family via a number of RNA recognition pathways that ultimately act to limit viral replication [1], [2]. Production of type I interferons (IFNs; IFN-α and IFN-β) by virus infected cells results in up-regulation of anti-viral IFN-stimulated genes (ISGs) and cytokines [3]. Infection of cells with the *flavivirus*, dengue virus (DENV) is recognised by the toll-like receptor-3 (TLR3), retinoic acid inducible gene–I (RIG-I) and melanoma differentiation associated gene-5 (MDA5) pathways to induce the IFN response [4], [5]. Microarray studies have shown up-regulation of ISGs, including viperin during DENV infection in cell lines and patient peripheral blood mononuclear cells (PBMC) [6], as well as during DENV infection of macaques in both macrophages and B-cells [7]. We and others have demonstrated that viperin is induced by infection with a number of diverse viruses as well as able to limit viral infection in most instances, including the first reported up-regulation of viperin in human cytomegalovirus (HCMV) infected cells [8], [9], [10]. Subsequently, viperin has been shown to have anti-viral actions in other viral infections such as hepatitis C virus (HCV), influenza virus, human immunodeficiency virus (HIV), sindbis virus (SINV), the *flaviviruses* Japanese encephalitis virus (JEV) and West Nile virus (WNV) [9], [10], [11], [12], [13], [14], [15] and more recently, Bunyamwera virus [16] and Chikungunya virus [17]. The roles and actions of viperin in these different viral infections appear diverse and multifaceted with anti-viral activity in some cases dependent on alterations to lipid rafts (influenza, [14]), membrane localisation (HCV, [9]), the radical *S*-adenosylmethionine (SAM) enzymatic activity of viperin (HIV, [10]), negated by viral proteins (JEV, [11]) and even an enhancing role under some conditions for HCMV [18]. Viperin also has anti-viral activity against DENV infection [6], [13] however the interaction of DENV and viperin has not been thoroughly investigated.

In this study we have further defined the induction of viperin and its mechanisms of anti-viral actions in DENV-2-infected cells, using an infectious DENV-2 *in vitro* replication model and including primary monocyte-derived macrophages (MDM) which represent a target cell type for DENV *in vivo*. Results show that DENV-2 infection induces viperin mRNA and protein, that expression of viperin is anti-viral, requiring the C-terminal but not N-terminal regions of viperin protein, and restricts DENV-2 infection by reducing viral RNA production. Viperin co-localised and interacted with DENV-2 CA, viral RNA and NS3 proteins. The interaction of viperin and NS3 but not membrane association, however, is necessary for viperins anti-viral actions. These results show both similarities and differences to our recent data suggesting that the anti-viral actions of viperin relate to its interaction with HCV NS5A and VAP-A in HCV replication complexes [9] and supports the growing evidence for both conserved and unique mechanisms of action of viperin against viral infections, even within the closely related *Flaviviridae* family of viruses.

## Materials and Methods

### Cells and virus stocks

Vero African green monkey kidney cells, A549, a human lung carcinoma cell line, Huh-7 and Huh-7.5 human hepatoma cells and primary monocyte-derived macrophages (MDM) were used for DENV-2 infection studies and maintained as previously described. Primary MDM were generated by adherence from PBMC that were isolated from voluntary blood donation at the Australian Red Cross Blood Service. Blood was provided anonymously and used with approval from the Southern Adelaide Clinical Human Research Ethics Committee. Infections utilised DENV-2, Mon601, a derivative of the New Guinea C strain [19] that was produced from *in vitro* transcribed RNA, transfected into BHK-21, baby hamster kidney cells, amplified in C6/36 insect cells and titred in Vero cells. Viperin shRNA and control cells were generated in Huh-7 cells, as previously described [9].

### DENV-2 infection and quantitation

Cells were infected at a multiplicity of infection (MOI) of 0.1 or 1 for cell lines and an MOI of 3 for MDM for 90 min at 37°C, as described previously [20], [21], [22]. At the indicated time points post infection (pi) cell culture supernatants were collected, clarified by centrifugation and stored at −80°C prior to performing a plaque assay in Vero cells as previously described [21].

### Western blot for viperin

MDM were generated and DENV-2 infected, as above and at 48 h pi cells lysed and lysates subjected to SDS-PAGE. Proteins were transferred to nitrocellulose membranes and probed for viperin (in house rabbit anti-viperin antibody, 1/1000 [12]) with detection of complexes with goat-anti-rabbit-HRP conjugate and chemiluminesence. Protein loading was normalised by re-probing filters for β-actin (anti-rabbit β-actin, 1/500, BioVision). Images were captured with a LAS-4000 imaging system (Fuji Corp) and quantitated using Carestream Molecular Imaging Software 5.02 (Carestream Health Inc).

### Plasmids and transfections

Wild type (WT) viperin and viperin mutant constructs were as described previously [9]. The DENV-2 NS3-GFP and pEPI–GFP CA constructs were a kind gift from Professor David Jans (Monash University, Australia). Viperin mCherry fusion proteins were created utilising pLenti6-mCherry; WT and viperin mutants were cloned in frame (*Xho*I/*Sac*II) into the construct using previously described primers [9]. Cell lines were transfected using FuGene6 (Roche, IN) as per manufacturer's instructions. The viperin coding region was cloned into the lentiviral vector pLenti6/V5-D-TOPO (Invitrogen, CA) and the control lentiviral plasmid pLenti6/V5-D-TOPO-tdTomato was obtained from Dr Yuka Harata-Lee (University of Adelaide, Adelaide). Infectious lentivirus was generated as previously described [23]. Primary MDM were transduced with tdTomato control or viperin expressing lentivirus for 90 min at 37°C and were DENV-2 challenged at 24 h post transduction.

### RNA extraction and RT-PCR

Total cellular RNA was isolated from cells using Trizol (Invitrogen), DNase treated and quantitated by spectrophotometry. For DENV-2 strand specific RT-PCR, 100 ng of denatured RNA was reverse transcribed at 37°C for 1 h with 20 pmol of DENV-2 specific primer (DENV5.1 or DENV3.2 [21]) attached to a 19mer long sequence (Tag) [24] and 10 U MMuLV RT (Promega, WI). The tagged DENV-2 cDNA was then subjected to real time PCR using SYBER Green PCR mix (Applied Biosystems, CA) and 20 pmole of each primer, Tag, DENV3.2 for negative (−ve strand) and DENV5.1 for positive (+ve strand), as previously described [21]. Real time PCR for viperin and the control gene RPLPO was performed as previously described on the ABI 7000 prism [12]. Primer sequences for IFIT1 were 5′ AACTTAATGCAGGAAGAACATGACAA and 5′ CTGCCAGTCTGCCCATGTG.

### Immunolabelling and co-localisation studies

Cells were cultured on gelatin coated glass coverslips, and fixed in either 1% (v/v) formaldehyde for MDM and HeLa, or acetone∶methanol (1∶1) for Huh-7 cells and stored at −20°C. Slides were washed in PBS, and the formaldehyde fixed cells permeabilised with 0.05% (v/v) IGEPAL before blocking in 4% (v/v) goat serum, 2% (v/v) human serum, 0.4% (w/v) bovine serum albumin (BSA) in Hanks buffered salts solution (Gibco BRL, NY). Cells were immunolabelled using mouse anti-DENV-2 (serotypes 1–4, Santa Cruz Biotechnology Inc, 1/100 dilution), a rabbit anti-viperin, a mouse anti-FLAG (Sigma, MO) or a mouse anti-DENV CA antibodies (a kind gift from Prof David Jans, Monash University, Australia). Immunoreactivity was detected with goat anti-mouse IgG-Alexa 488, a goat anti-rabbit IgG-Alexa 647 or a goat anti-mouse IgG-Alexa 555 secondary antibodies (Molecular probes, CA). Nuclei were labelled with Hoechst 33342 (Molecular Probes, CA). BODIPY 493/503 (Invitrogen) was prepared as a stock solution of 1 mg/ml in ethanol. Fluorescence was visualised by confocal laser scanning microscopy (Biorad Radiance 2100 or Leica SP5 Spectral Confocal Microscope). For some experiments, quantification of intensity of immunofluorescence labelling was performed using ImageJ software (National Institutes of Health). The mean grayscale value was obtained for each channel for all cells where the image plane passed through the nucleus and excluding any cells at edge of the image and clusters of overlapping cells. Thresholds for detection of DENV-2 immunoreactivity were set at a grayscale value of three standard errors of the mean above the mean grayscale value measured in mock infected cells.

### Immunoprecipitation

293T cells were transfected with pLenti6/V5-D-TOPO-viperin for 3 h then allowed to recover for 2 h prior to infection with DENV-2 at an MOI = 3. At 24 h pi cells were lysed (10 mM Tris, pH 7.5, 100 mM NaCl, 0.5% (v/v) Triton X-100+complete mini protease inhibitors [Roche]), lysates clarified and incubated for 1 hr with rabbit anti-FLAG antibody. Complexes were recovered with protein A-sepharose, washed six times (10 mM Tris, pH 7.5, 100 mM NaCl, 0.05% [v/v] Triton X-100+protease inhibitors) and resuspended in water. Precipitates were analysed for proteins by western blot and total DENV-2 RNA by RT-PCR with DENV5.1 and DENV3.2 primers, as described above with the exception that the reverse transcription step was non-primer directed.

### Fluorescence Energy Resonance Transfer (FRET) analysis

Acceptor photobleaching was carried out as previously described in [9] with the use of GFP and mCherry tagged protein constructs. Pre and post-bleaching images were aligned using ImageJ and the difference in fluorescence (DIF) analysed in 5–10 regions of each cell where lipid droplets and/or cytoplasmic stained structures were positive for both proteins. At least 10 different cells in each of at least two independent experiments were analysed to ensure reproducibility. Negative slides were prepared by imaging cells with only the donor molecule present and treated in parallel photobleaching experiments.

### Statistical analysis

Student t-tests were utilised to analyse the distributions of 2 normally distributed data sets and experiments were performed a minimum of three times, in triplicate or duplicate. Statistical analysis was performed using SPSS 10.

## Results

### Viperin mRNA and protein is induced following DENV-2 infection

**Figure 1:**
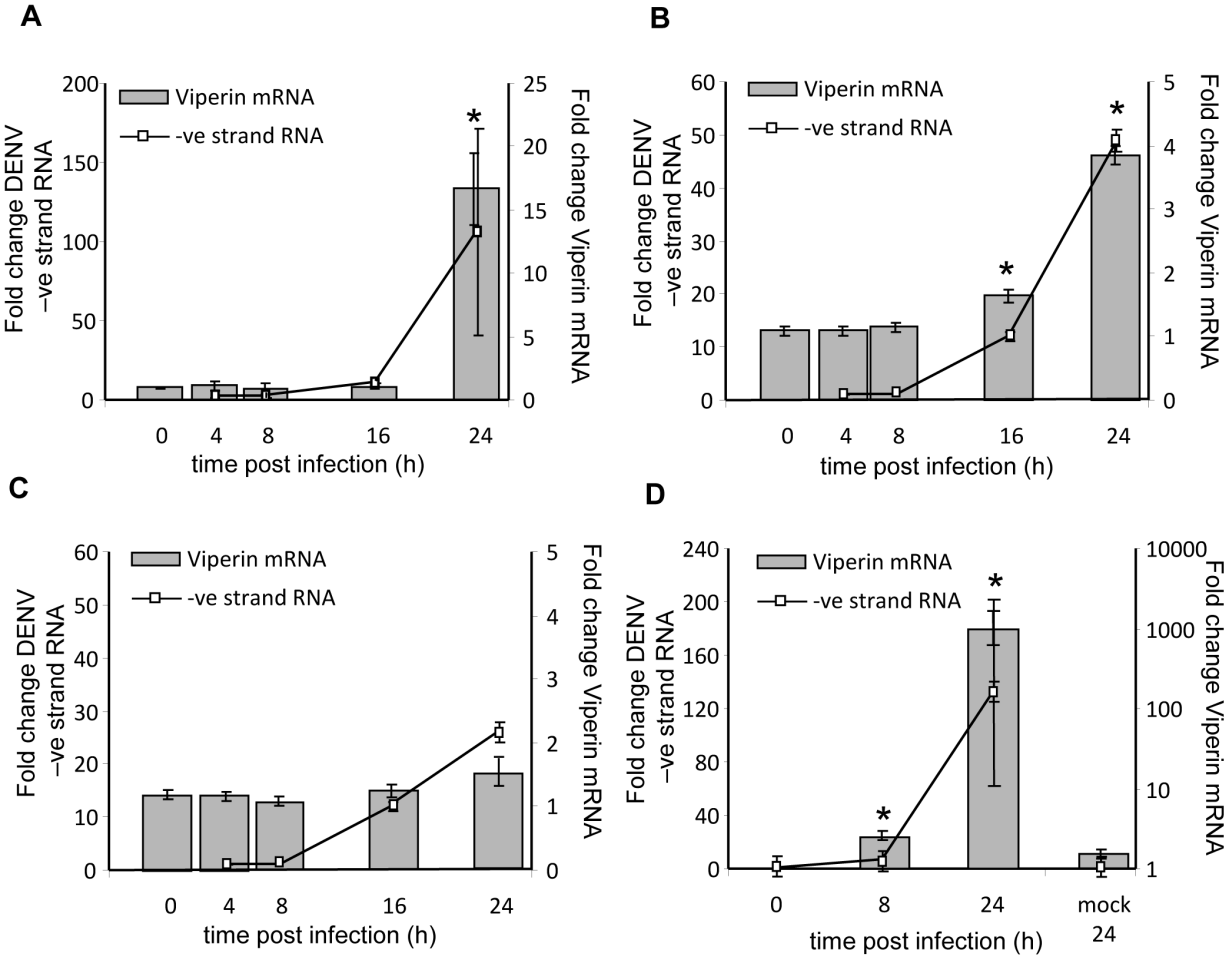
Viperin mRNA is induced in DENV-2 infected cells. Cells were infected with DENV-2 (MOI = 1 or MOI = 3 for MDM) and at various time points pi intracellular RNA was extracted and viperin mRNA and DENV −ve strand RNA quantitated by real time RT-PCR. Results were normalised against control RPLPO mRNA levels and expressed as fold change. Values represent average ± SEM (n = 3). (**A**) A549; (**B**) Huh-7; (**C**) Huh-7.5; (**D**) MDM. * Significantly different in comparison to 0 h time point, p<0.05.

As mentioned previously, a number of viruses are able to induce viperin expression, and to extend these observations we infected cell lines and primary MDM with DENV-2. Cells were lysed at the indicated time points pi, RNA extracted and analysed by RT-PCR for viperin and DENV-2 negative strand (−ve) RNA, which is a marker of productive DENV-2 replication. Viperin mRNA was significantly induced in DENV-2 infected human cell lines with approximately 25 fold induction in A549 lung carcinoma cells (Figure 1A) and a lesser 4 fold increase in Huh-7 hepatoma cells co-incident with high level DENV-2 −ve strand RNA production (Figure 1B). In contrast viperin mRNA was not increased following DENV-2 infection of Huh-7.5 cells, a cell line which is defective in dsRNA signalling via a mutation in the pathogen-recognition receptor RIG-I [25] (Figure 1C). Up-regulation of viperin by DENV-2 infection was also demonstrated in primary MDM, with a much greater, approximately 1000 fold induction of viperin mRNA at 24 h pi (Figure 1D).

**Figure 2:**
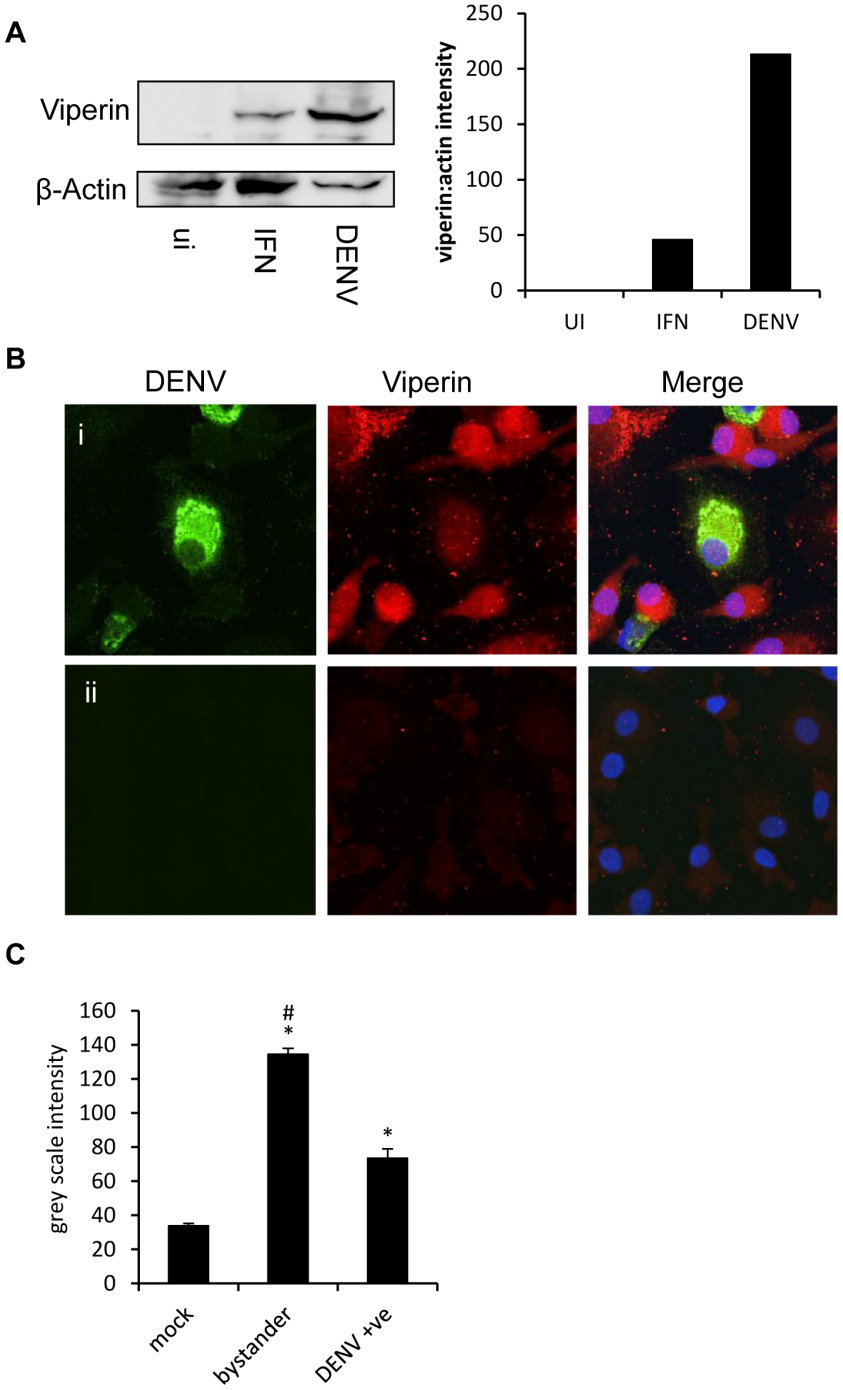
Viperin protein is induced in DENV-2 infected cells. **A.** Primary MDM were left uninfected, treated with 500 U/ml IFN-α or DENV-2 infected. At 48 h pi cells were lysed and viperin protein analysed by western blot. Blots were re-probed for β-actin and images visualised by chemiluminesence. Images were quantitated using Carestream Molecular Imaging Software and viperin signal normalised against β-actin. **B.** Primary MDM were DENV-2 (i) or mock (ii) infected and at 24 h pi were fixed and immunostained for viperin and DENV, with detection of stained complexes with anti-rabbit 647 (red) and anti-mouse 488 (green), respectively. Nuclei were stained with Hoechst (blue) and images collected by confocal microscopy. **C.** Immunolabeling for viperin was quantitated in cells from mock-infected MDM and compared with antigen negative bystander and DENV-2 antigen positive cells of the DENV-2 infected MDM cultures. Values represent average ± SEM. (n = 111 mock; 27 DENV-antigen positive; 136 DENV-antigen negative bystander cells). * = significantly different, p<0.05, Students unpaired t-test. Results of a single experiment are shown which was replicated.

We next assessed up-regulation of viperin protein in MDM since these showed the most significant change in viperin mRNA. Cells were DENV-2 infected, lysed and analysed for viperin by western blot with IFN-α treated cells used as a positive control. Results show increased levels of viperin protein in DENV-2 infected primary MDM, at levels greater than that induced by IFN alone (Figure 2A). We further characterised viperin protein in DENV-2-MDM by confocal microscopy. As can be seen in Figure 2B, at 24 h pi viperin was elevated in DENV-2 infected compared with mock infected MDM. Interestingly MDM positive for DENV antigen displayed reduced amounts of viperin protein, whereas DENV-antigen negative bystander cells (indicated via arrows, Figure 2B, upper panel) were shown to express significantly increased levels of viperin. The intensity of viperin staining in these different populations was quantitated. Results support the visual up-regulation of viperin in DENV-2 compared to mock infected cells, but most predominantly in the antigen negative, bystander cells of the DENV-2 infected population (Figure 2C).

### Viperin is anti-viral against DENV-2 infection *in vitro*

**Figure 3:**
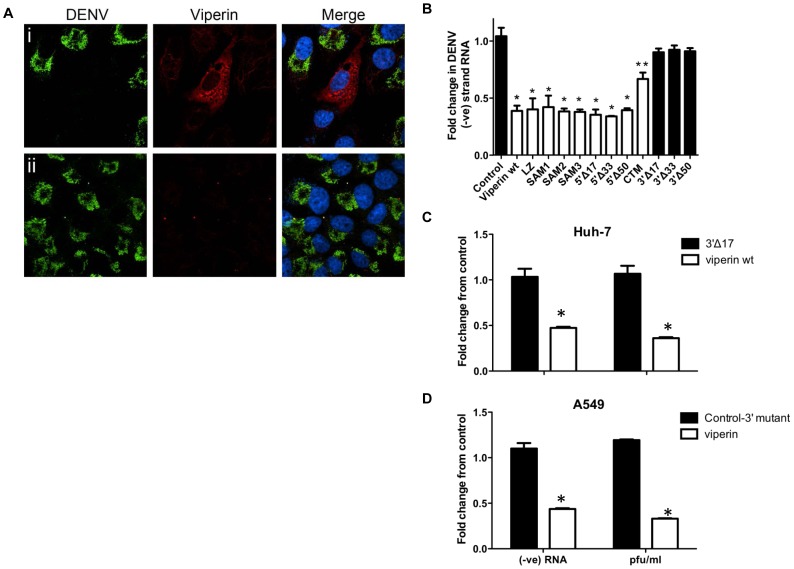
Viperin is anti-viral against DENV-2 and requires C-terminal regions of the protein. (**A**) HeLa cells were transfected with either a viperin-FLAG expression plasmid (**i**) or a control vector (**ii**) and at 24 h post transfection infected with DENV-2 (MOI = 1). At 24 h pi cells were fixed and immunolabelled with anti-FLAG (viperin) and anti-dsRNA antibodies with detection of stained complexes with anti-rabbit 647 (red) and anti-mouse 488 (green), respectively. Nuclei were stained with Hoechst (blue) and images collected by confocal microscopy. (**B**) Huh-7 cells were transfected to express WT viperin or viperin mutants and at 24 h post transfection infected with DENV-2 (MOI = 0.1). 24 h pi RNA was extracted and DENV-2 −ve strand PCR quantitated by real-time RT-PCR. Results were normalised against control RPLPO mRNA levels and expressed as fold change. Values represent average ± SEM (n = 3). * = significantly different to no viperin control, ** = significantly different to no viperin control and WT viperin, p<0.05, Students t-test. Similar experiments to (B) were performed in (**C**) Huh-7 or (**D**) A549. Cells were transfected using WT viperin or a 3′Δ17 viperin expression construct and infected as in (B). Supernatant was sampled and analysed for infectious virus release by plaque assay and RNA extracted from infected cells and DENV −ve strand RNA quantitated by real time RT-PCR. Results were normalised against control RPLPO mRNA levels and expressed as fold change relative to 3′Δ17 viperin control. Values represent average ± SEM (n = 3). * = significantly different to WT viperin, p<0.05, Students unpaired t-test.

Ectopic expression of viperin has been previously shown to inhibit DENV infection using virus and reporter virus-like particles *in vitro* [6], [13], however neither the anti-viral mechanism(s) or the interaction with viperin has been fully explored. Here we first transfected HeLa cells with plasmid to express WT viperin and at 24 h post transfection infected with DENV-2. Cells were fixed 24 h pi and immunolabelled for dsRNA and viperin. Results are suggestive of anti-DENV activity of viperin, with individual cells expressing viperin harbouring either no or very little DENV-2 RNA (Figure 3A). We next quantitated this potential anti-DENV-2 activity of viperin using a panel of viperin mutants that have previously been used to investigate viperins anti-HCV activity [9]. Although little is known about the structure/function relationship of viperins anti-viral activity, recent work by us and others has demonstrated that both the localisation of viperin to the ER membrane through its N-terminal amphipathic helix, as well as its C-terminal residues are essential for its ability to limit the replication of HCV [9], [26]. Transient expression of WT viperin in Huh-7 cells significantly inhibited DENV-2 −ve strand RNA levels by up to 61% (Figure 3B). A significant reduction of DENV-2 −ve strand RNA was also observed for cells transfected with viperin mutants in the SAM (SAM 1-3) domain, leucine zipper (LZ) and N-terminal deletions from 17 and up to 50 amino acid residues, suggesting that these regions play no role in the anti-viral activity of viperin (Figure 3B). In contrast, C-terminal deletions, as small as 17 amino acids, completely abolished the anti-DENV-2 activity of viperin (Figure 3B). These C-terminal deletion mutants have previously been shown in our laboratory to retain the same expression level and localisation as WT viperin [9]. Mutation of the single C-terminal residue of viperin (CTM) partly abrogated the anti-DENV-2 activity of WT viperin, although compared with the no viperin control the CTM still produced a significant reduction in DENV-2 −ve strand RNA levels (Figure 3B). These results highlight the importance of the C-terminal end of viperin for anti-viral activity and the C-terminal, 3′Δ17 mutant is used as a control in subsequent experiments.

Huh-7 or A549 cells were transfected to transiently express WT or the C-terminal 3′Δ17 mutant viperin lacking anti-viral activity, infected with DENV-2 and were analysed at 24 h pi (Figure 3C and D respectively). Results confirmed a significant reduction in both infectious virus release as determined by plaque assay of media from infected cells and production of −ve strand RNA induced by WT but not 3′Δ17 viperin expression in these two different cell types.

### Viperin is anti-viral against DENV-2 in primary human cells

**Figure 4:**
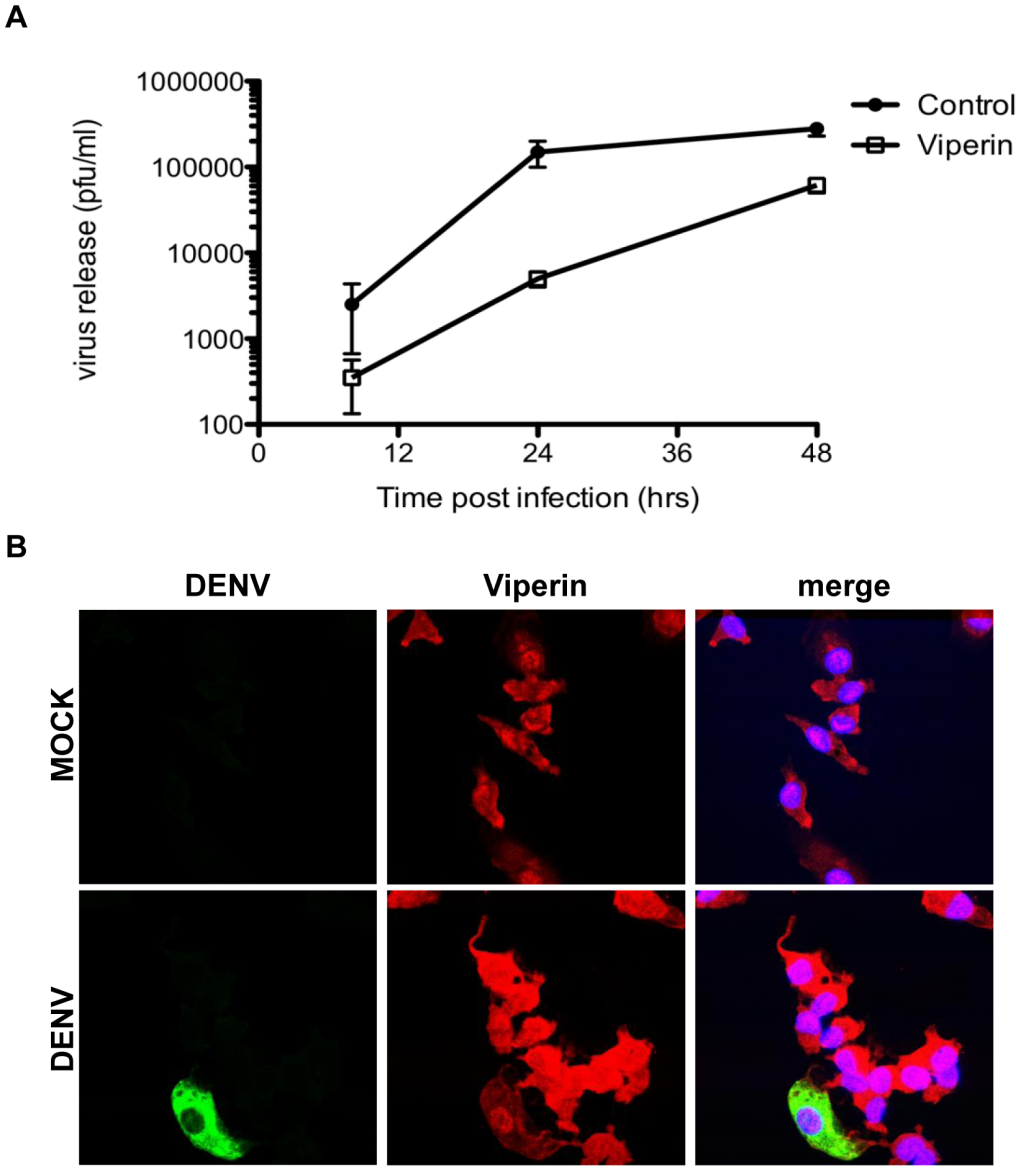
Viperin is anti-viral in primary MDM. Primary MDM were generated from peripheral blood and transduced with lentiviral particles expressing control td-Tomato or WT viperin. At 24 h post transduction, cells were infected with DENV-2 (MOI = 3). (**A**) Supernatant was sampled and infectious virus release quantitated by plaque assay. Values represent average ± SEM (n = 3). * p<0.001; (**B**) Viperin lenti-transduced MDM were DENV-2 or mock infected and at 48 h pi cells were fixed and immunolabelled for viperin and DENV with detection of complexes with Alexa-647 (red) and Alexa-488 (green), respectively. Nuclei were stained with Hoechst (blue) and images collected by confocal microscopy.

An important cell type for DENV infection *in vivo* are cells of the monocyte-macrophage lineage. Additionally, these cells are major contributors to the IFN response. As such we have analysed the anti-viral actions of viperin in primary MDM. Given the difficulty in transfecting MDM, we expressed viperin via lentivirus-mediated transduction. MDM were transduced with a td-Tomato-red fluorescent protein control or viperin encoding lentivirus expression vector, infected with DENV-2 and infection analysed. Results show a significant reduction in infectious virus release from lentivirus-viperin transduced MDM compared with lentivirus td-Tomato transduced control MDM, with a significant 30 and 4 fold decrease seen at 24 and 48 h pi respectively (the 8 h time point is considered a measure of input virus and is not significantly different between control and viperin transduced cells) (Figure 4A). At 48 h pi cells were fixed and immunostained for viperin and DENV-2 antigens, followed by confocal microscopy. Enumeration of >600 cells from 10 different fields and two different infections showed a significant reduction in the number of DENV-2 antigen +ve cells in viperin compared with tdTomato transduced cells (images not shown, 5.9%±0.8 vs 9.5%±0.6, p<0.05, Students unpaired t-test). Additionally, we observed dramatically higher levels of viperin protein in the DENV-2 infected cells compared with mock-infected viperin-lentivirus transduced cells (Figure 4B). Further, this up-regulation of viperin was again only observed in the DENV-2 antigen −ve bystander cells of this population (Figure 4B). This likely represents up-regulation of endogenous viperin, as demonstrated previously in DENV-2 infected MDM (Figure 2B).

### Knockdown of viperin *in vitro* increases DENV-2 replication

**Figure 5:**
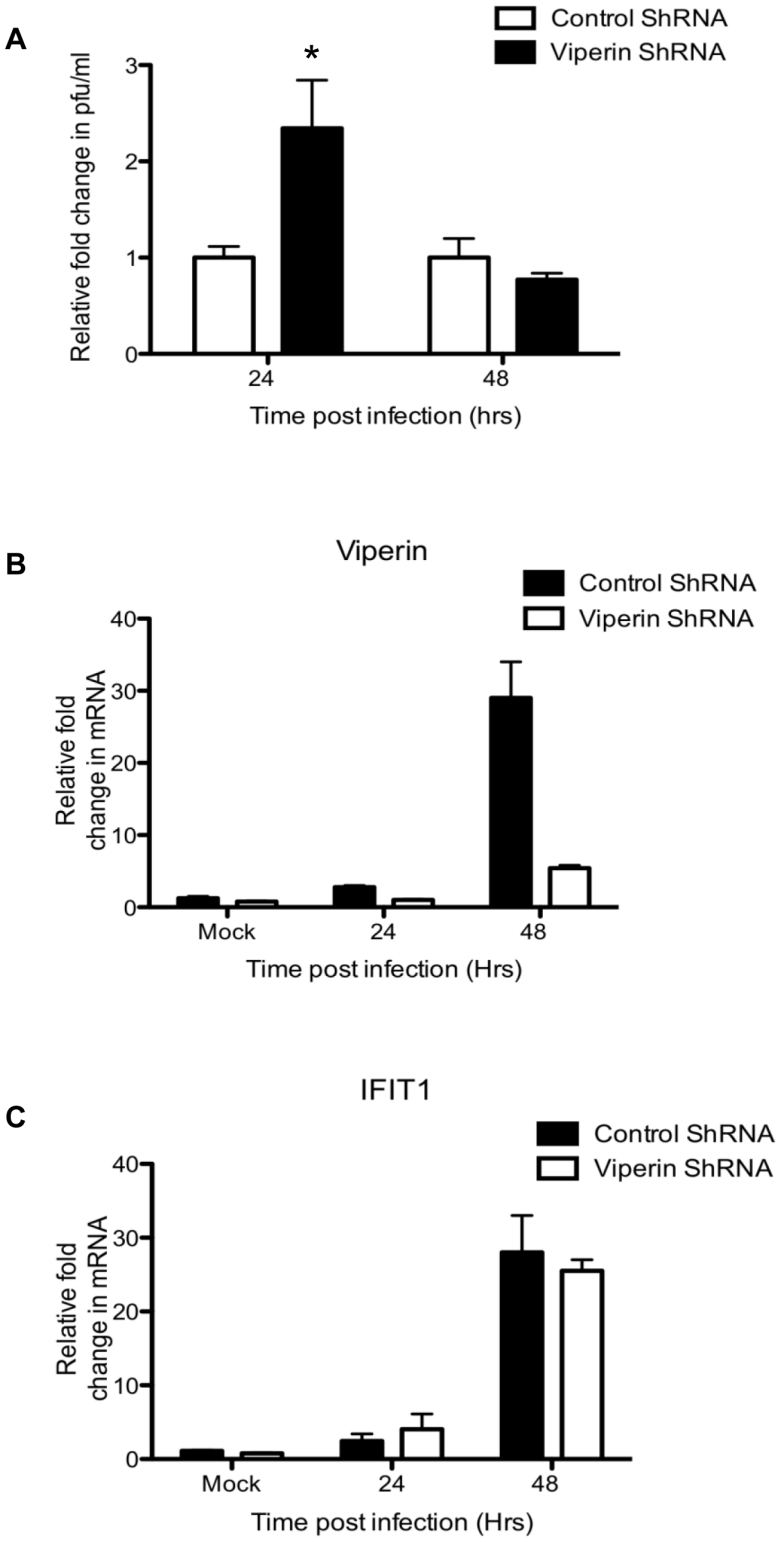
Induction of viperin is needed to restrict early viral replication. Viperin shRNA or control shRNA expressing Huh7 cells were infected with DENV-2 (MOI = 0.1). (**A**) Supernatant was sampled and infectious virus release quantitated by plaque assay. Values represent average ± SEM (n = 3); At the indicated time point pi cells were lysed, RNA extracted and analysed by real time RT-PCR for (**B**) viperin mRNA; (**C**) IFIT1 mRNA. Values represent average ± SEM (n = 4). Results were normalised against control RPLPO mRNA levels and expressed as fold change relative to mock infected cells. * = significant at p<0.05, Students unpaired t-test.

We next assessed the requirement for induction of viperin to restrict DENV-2 replication using a well characterised viperin shRNA Huh-7 cell line. Cells were DENV-2-infected at a lower MOI (0.1) to avoid DENV-induction of viperin mRNA, as in Figure 1, potentially overwhelming the capacity of the viperin shRNA. DENV-2 infection of viperin shRNA cells resulted in a significant, approximately 2 fold enhancement of infectious DENV-2 release at 24 h pi compared with control shRNA expressing cells (Figure 5A). By 48 h pi, infectious virus release was comparable between viperin shRNA and control shRNA expressing cells, possibly due to enhanced cytopathic effects in viperin shRNA cells associated with the earlier and higher level of DENV-2 replication, although this was not specifically quantitated. DENV-2-infection did not induce viperin mRNA in viperin shRNA expressing cells at 24 h pi (Figure 5B), although expression was detected at 48 h pi in some instances. In all cases, the unrelated ISG, IFIT1 mRNA, utilised as a control, was induced to comparable levels in both DENV-2-infected viperin shRNA and control shRNA expressing cells, demonstrating effective induction of other anti-viral responses in the absence of viperin (Figure 5C).

### Viperin reduces early viral RNA production but not viral entry

**Figure 6:**
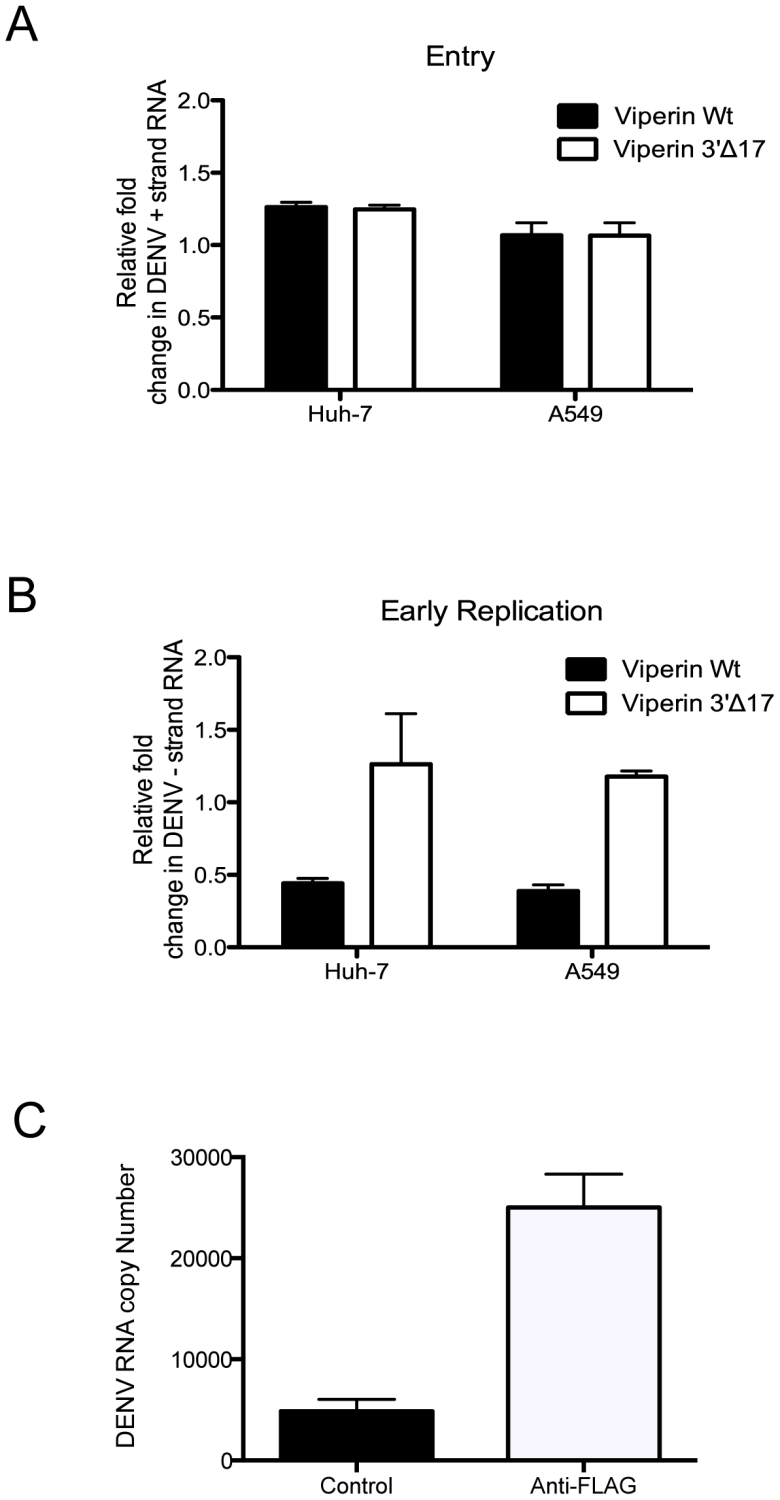
Viperin inhibits early DENV-2 replication and associates with DENV-2 RNA. A549 or Huh-7 cells were transfected with WT and 3′Δ17 viperin control and at 24 h post transfection infected with DENV-2 (MOI 0.1). (**A**) Immediately following infection cells were washed twice, trypsinised, lysed and DENV-2 +ve strand RNA quantitated by real time RT-PCR; (**B**) At 6 h pi RNA was extracted from infected cells and DENV-2 −ve strand RNA quantitated by real time RT-PCR; RT-PCR results were normalised against RPLPO mRNA levels. Values represent average ± SEM (n = 3), * p<0.05, Students unpaired t-test, (**C**) 293T cells were transfected with viperin-FLAG, IPed with FLAG antibody or no antibody control and precipitates analysed for DENV-2 RNA by RT-PCR. Values represent average ± SEM (n = 4), * p<0.05, Students unpaired t-test.

The lower levels of DENV-2 −ve strand RNA and viral release observed in HeLa, Huh-7 cells and primary MDM following infection of viperin expressing cells could be consistent with restriction of DENV-2 entry. To investigate this possibility Huh-7 and A549 cells were transfected to express viperin and following DENV-2 infection cells were immediately lysed and +ve strand DENV-2 RNA, indicative of intracellular genomic input RNA, quantitated by RT-PCR. Results showed no difference in intracellular levels of +ve strand DENV-2 RNA between cells transfected to express viperin and the inactive viperin C-terminal mutant, 3′Δ17 (Figure 6A). Additionally, DENV-2-infections were performed as above and cells lysed at 6 h pi and −ve strand DENV-2 RNA quantitated. Results demonstrated a significant reduction in the intracellular level of DENV-2 −ve strand RNA at this early time point in both A549 and Huh-7 cells transfected with viperin (Figure 6B), demonstrating a post-entry restriction in early DENV-2 RNA replication.

Our prior studies with HCV and viperin have demonstrated a requirement for the anti-viral actions of viperin mediated through lipid droplet and replication complex localisation and association with NS5A [9]. We next assessed the ability of viperin to associate with DENV-2 replication complexes by immunoprecipitation (IP). 293-T cells were transfected to express FLAG-tagged viperin, infected with DENV-2 then at 24 h pi cells lysed and IPed with anti-FLAG antibody. Precipitates were analysed for the presence of total DENV-2 RNA by RT-PCR. Results demonstrate successful co-precipitation of DENV-2 RNA with FLAG-antibody (Figure 6C). Concurrent analysis of precipitates by western blot, however failed to detect co-precipitated DENV-2 NS3 protein (data not shown).

### Viperin co-localises with both DENV-2 CA and NS3

**Figure 7:**
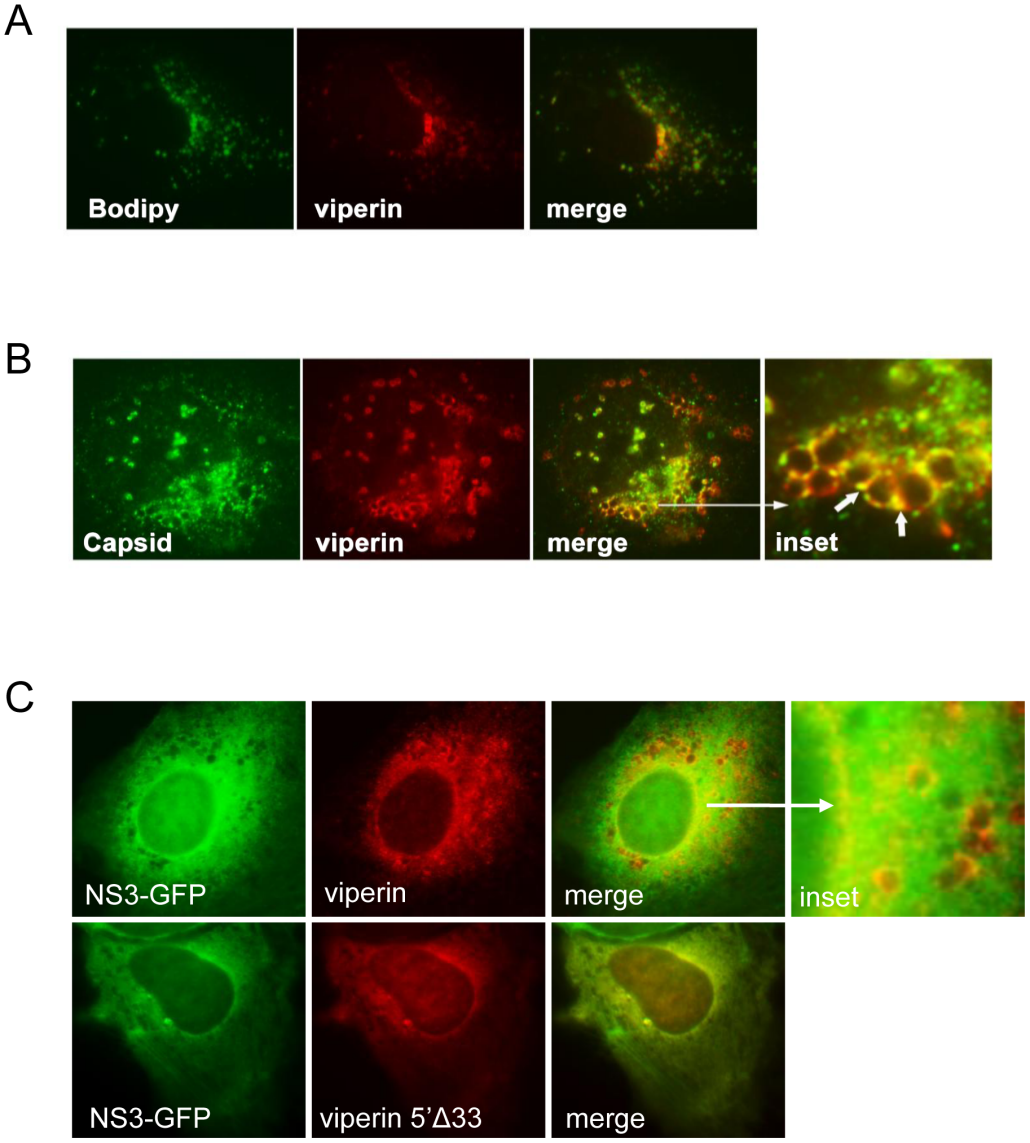
Viperin co-localises at lipid droplets and with DENV-2 CA and NS3. Huh-7 cells were transfected to express viperin-FLAG, DENV-2 infected (MOI 1) and at 24 h pi immunolabelled for viperin (anti-FLAG) and DENV, and examined via confocal microscopy for (**A**) viperin (red) using Alexa555 and BODIPY (green); (**B**) viperin (red) using Alexa555 and DENV-2 CA (green) using Alexa488. (**C**) **WT** or 5′Δ33 N-terminally FLAG tagged viperin and DENV-2 NS3-GFP (green) were transfected into Huh-7 cells and immunolabelled for viperin with detection of complexes with Alexa-555 (red).

The above data suggests the association of viperin with complexes containing DENV RNA (ie. replication complexes). Such complexes reportedly are also associated with cellular membrane structures and DENV-2 NS3 protein [27], [28]. The maintenance, however of the anti-DENV-2 activity of viperin containing N-terminal deletion mutants suggests that the ability of viperin to associate with membranes is not required for its restriction of DENV-2 infection (Figure 3A). We thus assessed the cellular localisation of viperin in DENV-2 infected cells. Viperin primarily localises to lipid droplets in Huh-7 cells as we have shown previously [9], and as can be seen in Figure 7A; this distribution remains unaltered in DENV-2 infected Huh-7 cells. The DENV capsid (CA) protein has also been demonstrated to localise to lipid droplets [29], [30] and consistent with these previous reports we observed partial co-localisation between DENV-2 CA and viperin at the interface of lipid droplet-like structures (white arrows, Figure 7B). Interestingly, viperin and the CA protein appear to coat the surface of the droplet at distinct loci, with small overlapping areas of co-localisation. Huh-7 cells were also co-transfected with a DENV-2 NS3-GFP expression plasmid and viperin. A clear co-localisation between DENV-2 NS3 and viperin is observed at the surface of lipid droplet-like structures as well as in distinct cytoplasmic loci (Figure 7C). The N-terminal viperin deletion mutant (Vip5′Δ33) which loses its ability to localise to lipid droplets [9], [31], but remains anti-viral against DENV-2 (Figure 3A), remained co-localised with DENV-2 NS3, although the pattern of localisation was solely cytoplasmic (Figure 7C). This observation demonstrates that viperin's anti-viral activities may be exerted through a possible interaction with NS3 but not necessarily at the lipid droplet interface.

### Viperin interacts with DENV-2 CA and NS3

As described above, although viperin co-precipitated DENV-2 RNA, IP experiments could not demonstrate co-precipitation of NS3 with viperin from either DENV-2 infected or NS3/viperin co-transfected cells (data not shown). These co-precipitation experiments are likely confounded by our observation of low levels of viperin protein in DENV-infected cells (Figure 2B) and low levels of DENV-2 antigens in viperin transfected cells (Figure 3A). Further, viperin is a lipid associated protein, which are notoriously difficult to extract and retain physiological protein-protein interactions.

**Figure 8:**
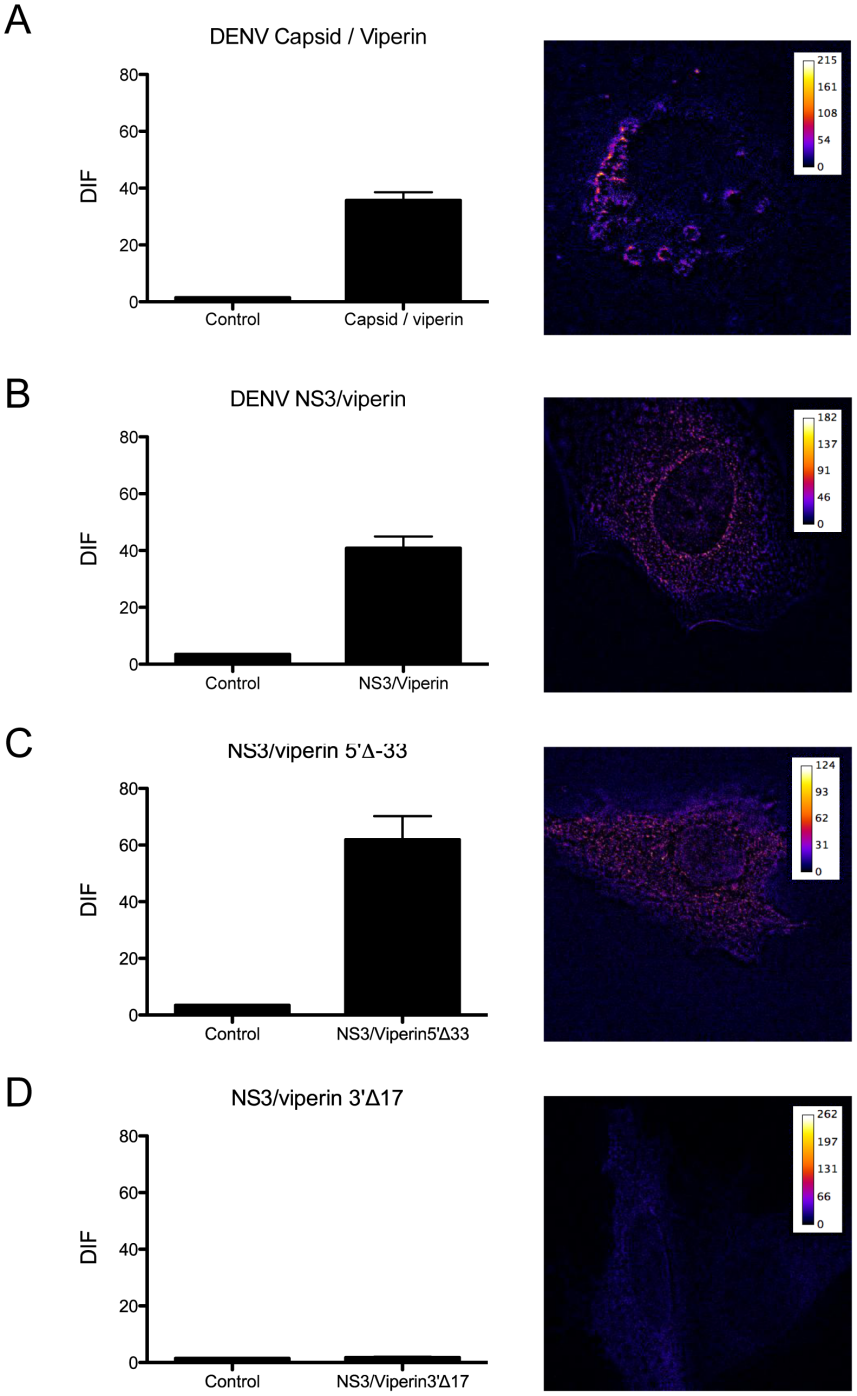
Viperin interacts with DENV-2 CA and NS3 protein. Huh-7 cells were co-transfected with a DENV-2 CA-GFP and viperin-mCherry expression vector (**A**), or DENV-2 NS3-GFP and expression plasmids for either viperin-mCherry (**B**), viperin 5′Δ33-mCherry (**C**) or viperin 3′Δ17-mCherry (**D**). Slides were analysed on a Zeiss Axioplan microscope and FRET determined by acceptor photobleaching. DIF was calculated from comparison of aligned pre and post-bleach images, from 5–10 regions per cell of at least 10 cells from 2 different experiments. Data are represented as average ± SEM with a significance of p<0.05 for (A), (B) and (C).

We therefore investigated the physical interaction of viperin with DENV-2 NS3 and CA by fluorescence energy resonance transfer (FRET). Huh-7 cells were transfected with expression plasmids for DENV-2 CA-GFP or DENV-2 NS3-GFP in conjunction with either mCherry N-terminally tagged viperin-WT, viperin 5′Δ33 or viperin 3′Δ17 and FRET acceptor photobleaching performed. Results show positive FRET for viperin-WT and the DENV-2 CA protein at the surface of the lipid droplet (Figure 8A) demonstrating an interaction of WT-viperin and CA proteins at this site. FRET analysis also demonstrated an interaction of DENV-2 NS3 and WT-viperin in distinct cytoplasmic foci (Figure 8B), similar to that seen in the confocal co-localisation studies of these two proteins (Figure 7C). No positive FRET was detected between DENV-2 NS3 and viperin surrounding lipid droplet like structures, despite our prior observation of co-localisation at these sites (Figure 7C). The ampipathic helix mutant (5′Δ33) of viperin, which retains its anti-DENV-2 activity, but has lost its membrane localisation ability, demonstrated a positive interaction by FRET analysis with DENV-2 NS3, once again at distinct cytoplasmic foci within the cells (Figure 8C). In contrast, the C-terminal viperin mutant, 3′Δ17, which has no anti-DENV-2 activity but maintains WT viperin localisation [9], showed no positive FRET with DENV-2 NS3 suggesting this protein is unable to interact with DENV-2 NS3. These results indicate that the C terminus of viperin mediates its anti-DENV-2 activity through an interaction with DENV-2 NS3 but does not require lipid droplet or membrane association.

## Discussion

Viperin is emerging as an important virus-induced ISG that can be up-regulated by both IFN-dependent and independent pathways and has a diverse array of anti-viral actions. Viperin can be induced in an IFN independent manner via IFN regulatory factor-1 (IRF-1), following infection with the RNA virus, vesicular stomatis virus (VSV) [32]. In contrast, SINV induction of viperin requires IFN but JEV induction of viperin occurs in an IFN-independent manner that requires IRF-3 and AP-1 [11]. In this study we show that viperin is induced early in DENV-2 infection and similar to our observation in HCV infected cells, does not occur in Huh-7.5 cells that are deficient in RIG-I [9]. IRF-3 and AP-1 are transcription factors downstream of RIG-I activation suggesting that the RIG-I pathway has an important role in induction of viperin, at least for the Flaviviridae members JEV, HCV and DENV. Furthermore, our studies in DENV-2-infected viperin shRNA cells suggest that the viperin already present or induced intracellularly in the DENV-2 infected cell acts to restrict or control DENV-2 infection in this initial target cell. Additionally our results from DENV-2-infected MDM show strong induction of viperin protein in DENV antigen negative bystander cells. This indicates that induction of viperin in these bystander cells, probably secondary to the release of IFN from the DENV-2 infected cell, is likely to also be important for restricting DENV-2 spread. Our observation of a far greater level of induction of viperin following DENV-2 infection compared with IFN stimulation of primary MDM suggests that induction of viperin by DENV-2, either in the initial DENV-2 infected cell or the uninfected bystander cell, occurs via factors other than IFN that are yet to be defined.

Viperin protein contains N-terminal ampipathic helical domains that direct viperin cellular localisation to the endoplasmic reticulum (ER) and lipid droplets [9], [31], [33]. The C-terminal portion of viperin is relatively unstructured and highly conserved amongst species; however its current function remains unknown. Previously we have demonstrated that the anti-viral actions of viperin are dependent on a number of functional domains of the viperin protein (i) the N-terminus for intracellular ER and lipid droplet localisation of viperin, (ii) the extreme C-terminus in the context of HCV replication [9], and (iii) the radical SAM domain in the context of HIV egress [10]. Using DENV-1 virus-like particles (VLP) and a luciferase reporter replicon system, a previous study has shown that viperin is induced by DENV-1 infection, inhibits DENV-1 RNA production and requires the N-terminal SAM1 domain of viperin [13]. This same study showed a similar induction of viperin, inhibition of RNA production and requirement for the viperin SAM1 domain and in part, residues within the first 50 amino acids of viperin during WNV infection. In contrast, our study observed anti-DENV-2 activity of viperin SAM1-4 mutants at levels comparable to WT viperin. The differences in the requirement for the viperin SAM1 domain seen in our current study compared with previous results with DENV-1 and WNV may be due to (i) the level of expression of viperin through use of a tet-induction system compared with the transient viperin transfection system in the current study; (ii) the analysis of different markers of infection with viperin SAM1 reducing infectious DENV-1 release [13] but in our study not DENV-2 −ve strand RNA; and/or (iii) the use of DENV-1 compared to DENV-2 herein. Regardless, studies clearly suggest that the anti-viral actions of viperin can be mediated by residues outside of the SAM1 domain, including the N-terminal 50 amino acid residues for WNV [13] and in our previous work with HCV, also the C-terminal regions of viperin [9]. Consistent with this requirement of the C-terminal region of viperin for anti-HCV activity, in our current study we have similarly defined anti-DENV activity to reside in the C-terminal 17 amino acids of viperin.

The specific regions of viperin necessary for anti-viral activity between various viruses differs, as does the biological effect of viperin during different virus infections. Viperin is reported to inhibit release of influenza virus by disruption of lipid rafts [14], to inhibit HIV egress [10] and to diminish viral protein production in HCMV infection [8]. In this study we show that viperin inhibits early post-entry DENV-2 RNA replication consistent with prior reported effects of viperin in inhibiting RNA replication in other Flaviviridae family members, HCV [9] and WNV [13]. In contrast, viperin is induced but is not anti-viral against the related *flavivirus*, JEV due to mechanisms of JEV that proteolyse and degrade viperin in infected cells [11]. However, contrary to earlier reports of anti-viral activity of viperin against HCMV, a recent study has shown that the vMIA protein of HCMV induces re-localisation of viperin from the ER to mitochondria, resulting in an increase in HCMV infection [18]. These contrasting effects of viperin suggest that its effects on viral infection are multifaceted, virus specific and involve multiple mechanisms of action including alterations in the subcellular localisation of viperin.

Viperin has been shown to localise to lipid droplets and the ER. Similarly we have shown here that viperin co-localises with the lipid droplet marker, BODIPY, in DENV-2 infected cells and thus the cellular localisation of viperin is unchanged during DENV-2 infection. The DENV CA localises to lipid droplets and preventing this CA-lipid droplet association reduces DENV RNA replication and infectious virus particle production [30]. While we confirm that viperin is able to co-localise and interact with the DENV-2 CA at lipid droplet-like structures (Figure 7B, 8B), our observation that viperin N-terminal mutants, which lose the ability to localise to lipid droplets, still retain substantial anti-DENV-2 activity suggests that viperin has significant anti-DENV-2 activities independent of its lipid droplet/CA association. We also demonstrate that viperin co-localises and interacts with the DENV-2-NS3 protein and co-precipitates with DENV-2 RNA, both of which are components of DENV replication complexes [27], [28]. The interaction of viperin and DENV-2 NS3 was independent of the N-terminal ampipathic helix, but reliant on the C-terminus of viperin. Further, the ability of viperin to co-localise and interact with DENV-2 NS3 correlated with anti-viral activity. We propose that viperin has anti-viral activity mediated by a C-terminus interaction with DENV-2 NS3 that reduces early RNA production by interfering with DENV-2 replication complexes. It remains to be determined whether this occurs solely through a direct interaction with the DENV-2 NS3 protein, or also through an intermediate pro-viral host cell factor, such as is the case for HCV, whereby viperin interacts with both NS5A and the pro-viral factor VAP-A [9], [26]. Currently, the only known pro-viral host factor for DENV that interacts with NS3 in the context of functional replication complexes is fatty acid synthetase (FASN) [34]. We feel FASN is an unlikely candidate as a target of viperin's actions since viperin and FASN exist in alternate cellular compartments (Figure S1).

In conclusion, this study has revealed further critical functions of viperin during DENV-2 replication and highlighted similarities and differences in the mechanisms of induction and in the anti-viral actions of viperin between DENV-2 and other medically important Flaviviridae. This data highlights the incredibly diverse anti-viral nature of viperin and the complexity of the viperin-virus interaction.

## Supporting Information

Figure S1 **Viperin does not co-localise with FASN.** Huh-7 cells were transiently transfected to express WT viperin-FLAG and at 24 h post transfection were immunolabelled for viperin (red, anti-FLAG) and FASN (green) with detection of complexes with Alexa-555 and Alexa-488, respectively.
(TIF)

## References

[1] Katze MG, He Y, Gale M Jr (2002) Viruses and interferon: a fight for supremacy. Nat Rev Immunol 2: 675–687. 12209136 10.1038/nri888

[2] Sen GC (2001) Viruses and interferons. Annu Rev Microbiol 55: 255–281. 11544356 10.1146/annurev.micro.55.1.255

[3] Munoz-Jordan JL, Fredericksen BL (2010) How flaviviruses activate and suppress the interferon response. Viruses 2: 676–691. 21994652 10.3390/v2020676 PMC3185611

[4] Nasirudeen AM, Liu DX (2009) Gene expression profiling by microarray analysis reveals an important role for caspase-1 in dengue virus-induced p53-mediated apoptosis. J Med Virol 81: 1069–1081. 19382257 10.1002/jmv.21486

[5] Nasirudeen AM, Wong HH, Thien P, Xu S, Lam KP, et al (2011) RIG-I, MDA5 and TLR3 synergistically play an important role in restriction of dengue virus infection. PLoS Negl Trop Dis 5: e926. 21245912 10.1371/journal.pntd.0000926 PMC3014945

[6] Fink J, Gu F, Ling L, Tolfvenstam T, Olfat F, et al (2007) Host gene expression profiling of dengue virus infection in cell lines and patients. PLoS Negl Trop Dis 1: e86. 18060089 10.1371/journal.pntd.0000086 PMC2100376

[7] Sariol CA, Munoz-Jordan JL, Abel K, Rosado LC, Pantoja P, et al (2007) Transcriptional activation of interferon-stimulated genes but not of cytokine genes after primary infection of rhesus macaques with dengue virus type 1. Clin Vaccine Immunol 14: 756–766. 17428947 10.1128/CVI.00052-07 PMC1951081

[8] Chin KC, Cresswell P (2001) Viperin (cig5), an IFN-inducible antiviral protein directly induced by human cytomegalovirus. Proc Natl Acad Sci U S A 98: 15125–15130. 11752458 10.1073/pnas.011593298 PMC64994

[9] Helbig KJ, Eyre NS, Yip E, Narayana S, Li K, et al (2011) The antiviral protein viperin inhibits HCV replication via interaction with NS5A. Hepatology 54: 1506–1517. 22045669 10.1002/hep.24542 PMC3207276

[10] Nasr N, Maddocks S, Turville SG, Harman AN, Woolger N, et al (2012) HIV-1 infection of human macrophages directly induces viperin which inhibits viral production. Blood 120: 778–788. 22677126 10.1182/blood-2012-01-407395

[11] Chan YL, Chang TH, Liao CL, Lin YL (2008) The cellular antiviral protein viperin is attenuated by proteasome-mediated protein degradation in Japanese encephalitis virus-infected cells. J Virol 82: 10455–10464. 18768981 10.1128/JVI.00438-08 PMC2573197

[12] Helbig KJ, Lau DT, Semendric L, Harley HA, Beard MR (2005) Analysis of ISG expression in chronic hepatitis C identifies viperin as a potential antiviral effector. Hepatology 42: 702–710. 16108059 10.1002/hep.20844

[13] Jiang D, Weidner JM, Qing M, Pan XB, Guo H, et al (2010) Identification of five interferon-induced cellular proteins that inhibit west nile virus and dengue virus infections. J Virol 84: 8332–8341. 20534863 10.1128/JVI.02199-09 PMC2916517

[14] Wang X, Hinson ER, Cresswell P (2007) The interferon-inducible protein viperin inhibits influenza virus release by perturbing lipid rafts. Cell Host Microbe 2: 96–105. 18005724 10.1016/j.chom.2007.06.009

[15] Tan KS, Olfat F, Phoon MC, Hsu JP, Howe JL, et al (2012) In vivo and in vitro studies on the antiviral activities of viperin against influenza H1N1 virus infection. J Gen Virol 93: 1269–1277. 22377585 10.1099/vir.0.040824-0

[16] Carlton-Smith C, Elliott RM (2012) Viperin, MTAP44, and protein kinase R contribute to the interferon-induced inhibition of Bunyamwera Orthobunyavirus replication. J Virol 86: 11548–11557. 22896602 10.1128/JVI.01773-12 PMC3486307

[17] Teng TS, Foo SS, Simamarta D, Lum FM, Teo TH, et al (2012) Viperin restricts chikungunya virus replication and pathology. J Clin Invest 122: 4447–4460. 23160199 10.1172/JCI63120 PMC3533538

[18] Seo JY, Yaneva R, Hinson ER, Cresswell P (2011) Human cytomegalovirus directly induces the antiviral protein viperin to enhance infectivity. Science 332: 1093–1097. 21527675 10.1126/science.1202007

[19] Gualano RC, Pryor MJ, Cauchi MR, Wright PJ, Davidson AD (1998) Identification of a major determinant of mouse neurovirulence of dengue virus type 2 using stably cloned genomic-length cDNA. J Gen Virol 79 (Pt 3) 437–446. 9519821 10.1099/0022-1317-79-3-437

[20] Carr JM, Hocking H, Bunting K, Wright PJ, Davidson A, et al (2003) Supernatants from dengue virus type-2 infected macrophages induce permeability changes in endothelial cell monolayers. J Med Virol 69: 521–528. 12601760 10.1002/jmv.10340

[21] Wati S, Li P, Burrell CJ, Carr JM (2007) Dengue virus (DV) replication in monocyte-derived macrophages is not affected by tumor necrosis factor alpha (TNF-alpha), and DV infection induces altered responsiveness to TNF-alpha stimulation. J Virol 81: 10161–10171. 17626094 10.1128/JVI.00313-07 PMC2045434

[22] Wati S, Rawlinson SM, Ivanov RA, Dorstyn L, Beard MR, et al (2011) Tumour necrosis factor alpha (TNF-alpha) stimulation of cells with established dengue virus type 2 infection induces cell death that is accompanied by a reduced ability of TNF-alpha to activate nuclear factor kappaB and reduced sphingosine kinase-1 activity. J Gen Virol 92: 807–818. 21148274 10.1099/vir.0.028159-0

[23] Eyre NS, Drummer HE, Beard MR (2010) The SR-BI partner PDZK1 facilitates hepatitis C virus entry. PLoS Pathog 6: e1001130. 20949066 10.1371/journal.ppat.1001130 PMC2951368

[24] Peyrefitte CN, Pastorino B, Bessaud M, Tolou HJ, Couissinier-Paris P (2003) Evidence for in vitro falsely-primed cDNAs that prevent specific detection of virus negative strand RNAs in dengue-infected cells: improvement by tagged RT-PCR. J Virol Methods 113: 19–28. 14500123 10.1016/s0166-0934(03)00218-0

[25] Sumpter R Jr, Loo YM, Foy E, Li K, Yoneyama M, et al (2005) Regulating intracellular antiviral defense and permissiveness to hepatitis C virus RNA replication through a cellular RNA helicase, RIG-I. J Virol 79: 2689–2699. 15708988 10.1128/JVI.79.5.2689-2699.2005 PMC548482

[26] Wang S, Wu X, Pan T, Song W, Wang Y, et al (2011) Viperin inhibits hepatitis C virus replication by interfering with binding of NS5A to host protein hVAP-33. J Gen Virol 93: 83–92. 21957124 10.1099/vir.0.033860-0

[27] Welsch S, Miller S, Romero-Brey I, Merz A, Bleck CK, et al (2009) Composition and three-dimensional architecture of the dengue virus replication and assembly sites. Cell Host Microbe 5: 365–375. 19380115 10.1016/j.chom.2009.03.007 PMC7103389

[28] Clum S, Ebner KE, Padmanabhan R (1997) Cotranslational membrane insertion of the serine proteinase precursor NS2B-NS3(Pro) of dengue virus type 2 is required for efficient in vitro processing and is mediated through the hydrophobic regions of NS2B. J Biol Chem 272: 30715–30723. 9388208 10.1074/jbc.272.49.30715

[29] Carvalho FA, Carneiro FA, Martins IC, Assuncao-Miranda I, Faustino AF, et al (2011) Dengue Virus Capsid Protein Binding to Hepatic Lipid Droplets (LD) Is Potassium Ion Dependent and Is Mediated by LD Surface Proteins. J Virol 86: 2096–2108. 22130547 10.1128/JVI.06796-11 PMC3302401

[30] Samsa MM, Mondotte JA, Iglesias NG, Assuncao-Miranda I, Barbosa-Lima G, et al (2009) Dengue virus capsid protein usurps lipid droplets for viral particle formation. PLoS Pathog 5: e1000632. 19851456 10.1371/journal.ppat.1000632 PMC2760139

[31] Hinson ER, Cresswell P (2009) The N-terminal amphipathic alpha-helix of viperin mediates localization to the cytosolic face of the endoplasmic reticulum and inhibits protein secretion. J Biol Chem 284: 4705–4712. 19074433 10.1074/jbc.M807261200 PMC2640954

[32] Stirnweiss A, Ksienzyk A, Klages K, Rand U, Grashoff M, et al (2010) IFN regulatory factor-1 bypasses IFN-mediated antiviral effects through viperin gene induction. J Immunol 184: 5179–5185. 20308629 10.4049/jimmunol.0902264

[33] Hinson ER, Cresswell P (2009) The antiviral protein, viperin, localizes to lipid droplets via its N-terminal amphipathic alpha-helix. Proc Natl Acad Sci U S A 106: 20452–20457. 19920176 10.1073/pnas.0911679106 PMC2778571

[34] Heaton NS, Perera R, Berger KL, Khadka S, Lacount DJ, et al (2010) Dengue virus nonstructural protein 3 redistributes fatty acid synthase to sites of viral replication and increases cellular fatty acid synthesis. Proc Natl Acad Sci U S A 107: 17345–17350. 20855599 10.1073/pnas.1010811107 PMC2951450

