# Supplementary figures and images for: Viperin Is Induced following Dengue Virus Type-2 (DENV-2) Infection and Has Anti-viral Actions Requiring the C-terminal End of Viperin
Source: PLoS Negl Trop Dis. 2013 Apr 18;7(4):e2178. doi: 10.1371/journal.pntd.0002178 (PMC3630087; doi:10.1371/journal.pntd.0002178)

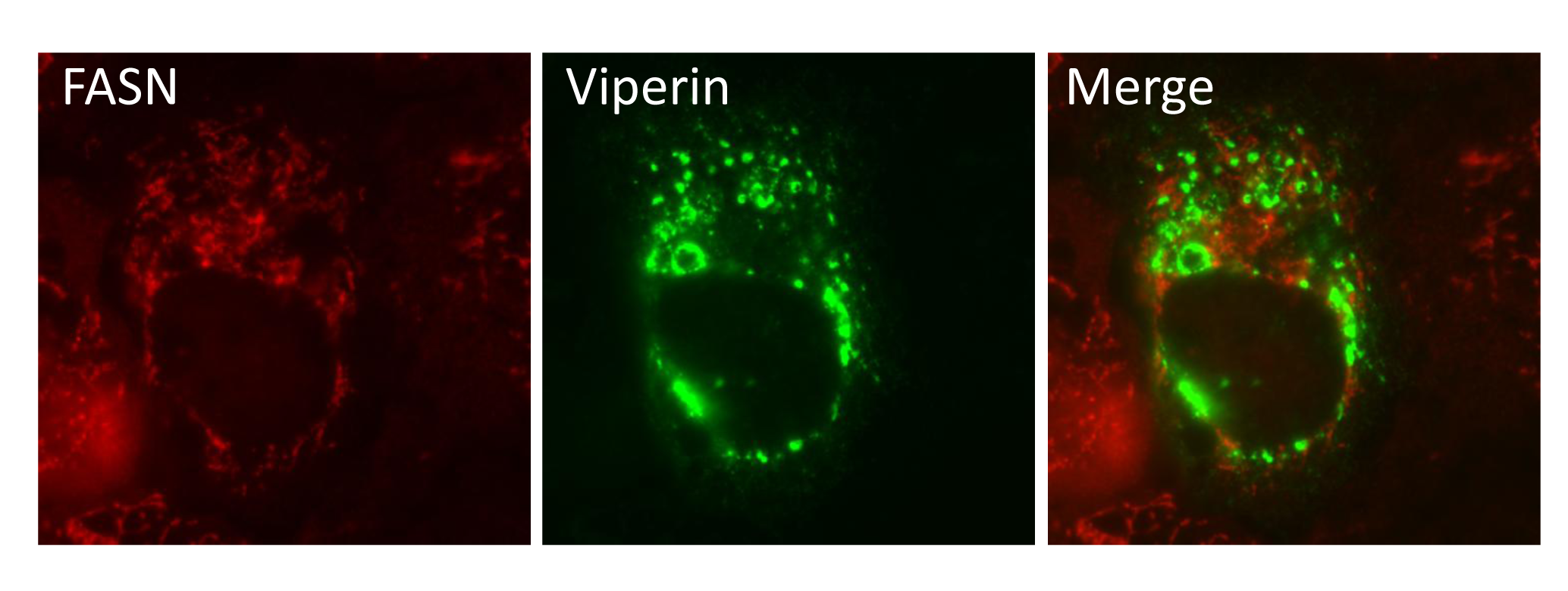

Supplement: Figure S1 — Viperin does not co-localise with FASN. Huh-7 cells were transiently transfected to express WT viperin-FLAG and at 24 h post transfection were immunolabelled for viperin (red, anti-FLAG) and FASN (green) with detection of complexes with Alexa-555 and Alexa-488, respectively. (TIF) [file pntd.0002178.s001.tif]
